# Protect Our Kids: a novel program bringing hemorrhage control to schools

**DOI:** 10.1186/s40621-021-00318-w

**Published:** 2021-09-13

**Authors:** Joseph Tobias, Aaron Cunningham, Kelsi Krakauer, Deepthi Nacharaju, Lori Moss, Carlos Galindo, Michael Roberts, Nicholas A. Hamilton, Kyle Olsen, Molly Emmons, Jim Quackenbush, Martin A. Schreiber, Beech S. Burns, David Sheridan, Benjamin Hoffman, Adrienne Gallardo, Mubeen A. Jafri

**Affiliations:** 1 Department of Surgery, Oregon Health & Science University, 3181 SW Sam Jackson Park Rd, Portland, OR 97239 USA; 2 School of Medicine, Oregon Health & Science University, Portland, OR USA; 3 Department of Pediatrics, Doernbecher Children’s Hospital, Oregon Health & Science University, Portland, OR USA; 4 Portland Public Schools, Portland, OR USA; 5 Portland Police Bureau, Portland, OR USA; 6 Department of Surgery, Division of Pediatric Surgery, Oregon Health & Science University, Portland, OR USA; 7 Division of Trauma, Critical Care and Acute Care Surgery, Department of Surgery, Oregon Health & Science University, Portland, OR USA; 8 Department of Emergency Medicine, Oregon Health & Science University, Portland, OR USA; 9 Division of Pediatric Surgery, Randall Children’s Hospital at Legacy Emanuel, Portland, OR USA

**Keywords:** Stop the Bleed, Hemorrhage control, Intentional mass casualty event, Active shooter event

## Abstract

**Background:**

Following the shooting at Sandy Hook Elementary School, the Hartford Consensus produced the Stop the Bleed program to train bystanders in hemorrhage control. In our region, the police bureau delivers critical incident training to public schools, offering instruction in responding to violent or dangerous situations. Until now, widespread training in hemorrhage control has been lacking. Our group developed, implemented and evaluated a novel program integrating hemorrhage control into critical incident training for school staff in order to blunt the impact of mass casualty events on children.

**Methods:**

The staff of 25 elementary and middle schools attended a 90-minute course incorporating Stop the Bleed into the critical incident training curriculum, delivered on-site by police officers, nurses and doctors over a three-day period. The joint program was named Protect Our Kids. At the conclusion of the course, hemorrhage control kits and educational materials were provided and a four-question survey to assess the quality of training using a ten-point Likert scale was completed by participants and trainers.

**Results:**

One thousand eighteen educators underwent training. A majority were teachers (78.2%), followed by para-educators (5.8%), counselors (4.4%) and principals (2%). Widely covered by local and state media, the Protect Our Kids program was rated as excellent and effective by a majority of trainees and all trainers rated the program as excellent.

**Conclusions:**

Through collaboration between trauma centers, police and school systems, a large-scale training program for hemorrhage control and critical incident response can be effectively delivered to schools.

## Background

Uncontrolled hemorrhage is the leading cause of preventable death after trauma in the United States (Berwick 2016). Although individual injury is the most common insult, intentional mass casualty events represent a steadily increasing threat. Between 2000 and 2018, 277 active shooter events killed 884 people and wounded 1546 (Federal Bureau of Investigation 2019). Twenty-one percent of these events took place in schools or universities.

Motivated by the Sandy Hook Elementary School shooting in 2012, the American College of Surgeons assembled the Joint Committee to Create a National Policy to Enhance Survivability from Intentional Mass Casualty and Active Shooter Events (Jacobs et al. 2015). The Committee’s efforts resulted in the Hartford Consensus, a landmark report that focuses on how the trauma system should address preventable death from hemorrhage in the prehospital setting. The pillars of the Hartford Consensus are earlier hemorrhage control and improved access to bleeding control kits (Jacobs et al. 2016). The need for wide-spread dissemination of these initiatives became clear several months following the Sandy Hook shooting in the 2013 Boston Marathon bombing. Medical providers, first responders and untrained bystanders rushed to aid victims with exsanguinating extremity injuries. Twenty-seven improvised but ultimately ineffective tourniquets were applied (King et al. 2015). Only one commercially designed tourniquet was used (King et al. 2015).

In response to the Hartford Consensus, the White House National Security Council launched the Stop the Bleed campaign in 2015. Stop the Bleed trains laypersons in hemorrhage control techniques, including applying direct pressure, packing wounds with gauze and placing tourniquets on model limbs. Training empowers bystanders to serve as immediate responders in bleeding emergencies and is predicated on recent wartime military experience showing that the prehospital use of tourniquets significantly decreases mortality in exsanguinating extremity injury (Kotwal et al. 2011).

Stop the Bleed is usually taught as a 90-minute course consisting of a didactic presentation followed by hands-on practice. It is likened to programs that teach medically unsophisticated bystanders first aid, cardiopulmonary resuscitation (CPR) and the use of automatic external defibrillators (AEDs). The effectiveness of training laypersons in hemorrhage control has been confirmed in observational studies and randomized controlled trials (Goralnick et al. 2018; Goolsby et al. 2015; Sidwell et al. 2018; Jayaraman et al. 2009; Ross et al. 2018). To date, Stop the Bleed has been taught to more than 1 million people in the United States, including to health care workers, police officers, school teachers and even to research scientists in Antarctica (American College of Surgeons 2020).

Through a systematic review of available data, the Pediatric Trauma Society released a position statement in 2017 supporting the use of tourniquets in children suffering exsanguinating extremity injury (Cunningham et al. 2018). This set a precedent to apply bystander hemorrhage control techniques to injured children. In response, our trauma system developed a partnership with local law enforcement to serve as a model for delivering Stop the Bleed to schools.

## Methods

Oregon is a small state with a population of 4.14 million residents, more than half of whom live in the Portland metropolitan area (United States Census Bureau 2019). Doernbecher Children’s Hospital at Oregon Health & Science University (OHSU) is a level I pediatric trauma center serving the entire state of Oregon, as well as southern Washington, northern California and western Idaho. Funded by the Pediatric Trauma Society and the Childress Institute for Pediatric Trauma, the Trauma Program at Doernbecher Children’s Hospital collaborated with the Portland Police Bureau (PPB) to integrate Stop the Bleed into Critical Incident Training at Portland Public Schools. Critical Incident Training by the PPB instructs school staff in responding to emergency situations to prevent entry of suspicious persons and maximize safety during active shooter events. The joint program was named Protect Our Kids.

Two teams comprising PPB school resource officers and doctors and nurses from OHSU, all trained in Stop the Bleed, visited 25 of 75 middle and elementary schools in the Portland metropolitan area over the course of 3 days prior to the start of the school year. A 90-minute joint course was held consisting of Critical Incident Training and Stop the Bleed. Participants began by watching a 30-minute video produced by the PPB, with instruction on how to cultivate situational awareness and perform options-based decision making during a critical incident requiring lockdown, shelter-in-place or evacuation. The video also served to introduce police personnel who are school resource officers and may respond during a critical incident. Thereafter, using the national Stop the Bleed curriculum developed by the American College of Surgeons, trainers equipped with a standardized PowerPoint presentation taught the ABCs of hemorrhage control: **A**lert 911, identify the source of **B**leeding and **C**ompress with manual pressure, wound packing or tourniquet application. This 30-minute didactic session was followed by 30 minutes of guided hands-on practice with model prosthetic limbs. Dedicated question-and-answer time was afforded during each didactic session and there was a ratio of less than or equal to eight participants per trainer during guided hands-on practice.

Schools received hemorrhage control kits, each containing gloves, shears, standard gauze, four tourniquets and Stop the Bleed instructional material. Perishable items, such as combat gauze, were excluded in order to give kits an indefinite shelf life. Kits were fabricated at roughly half the cost ($122/kit with four tourniquets) of those currently available from the American College of Surgeons ($650/kit with eight tourniquets) (American College of Surgeons 2020). At the conclusion of the course, participants completed a four-question survey using a ten-point Likert scale to assess the quality of training.

## Results

**Fig. 1:**
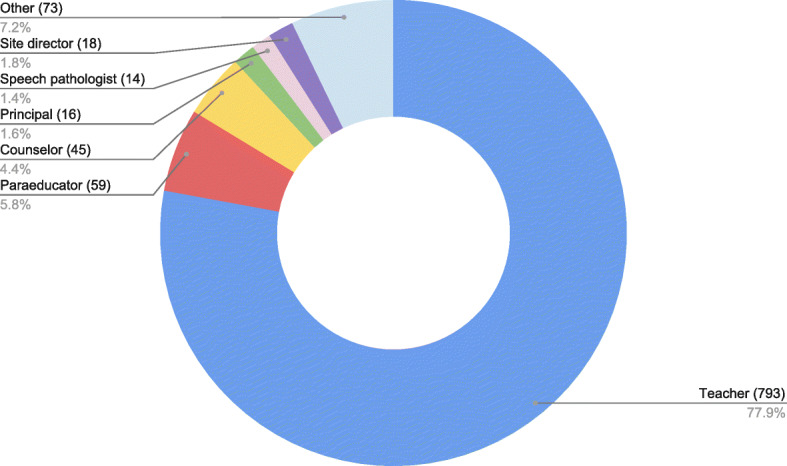
One thousand eighteen program trainees by occupation, including teachers, paraeducators such as teacher’s aides and classroom assistants, school counselors, speech pathologists, site directors for after school and extracurricular programming and other school staff such as clerks and child care providers

**Fig. 2:**
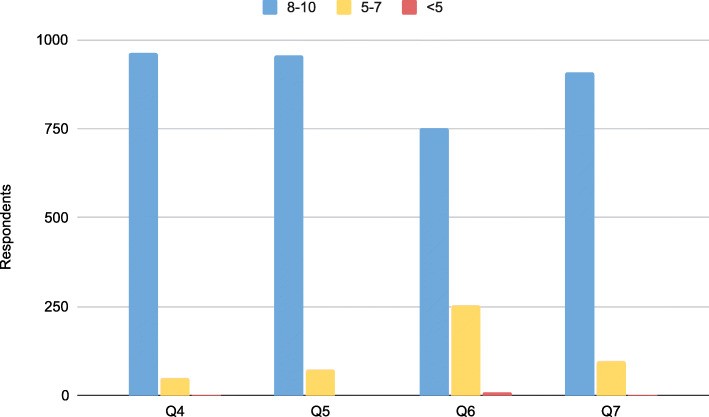
Trainee survey responses after completion of the program: questions 1–3 captured demographic data while questions 4–7, above, evaluated the program’s importance and effectiveness on a 10-point Likert scale: Q4 – how important is the program to *you*?. Q5 – how important is the program to the *public*?. Q6 – how prepared do you feel in a bleeding emergency?. Q7 – how effective is the program in teaching lifesaving skills during a critical incident?

**Fig. 3:**
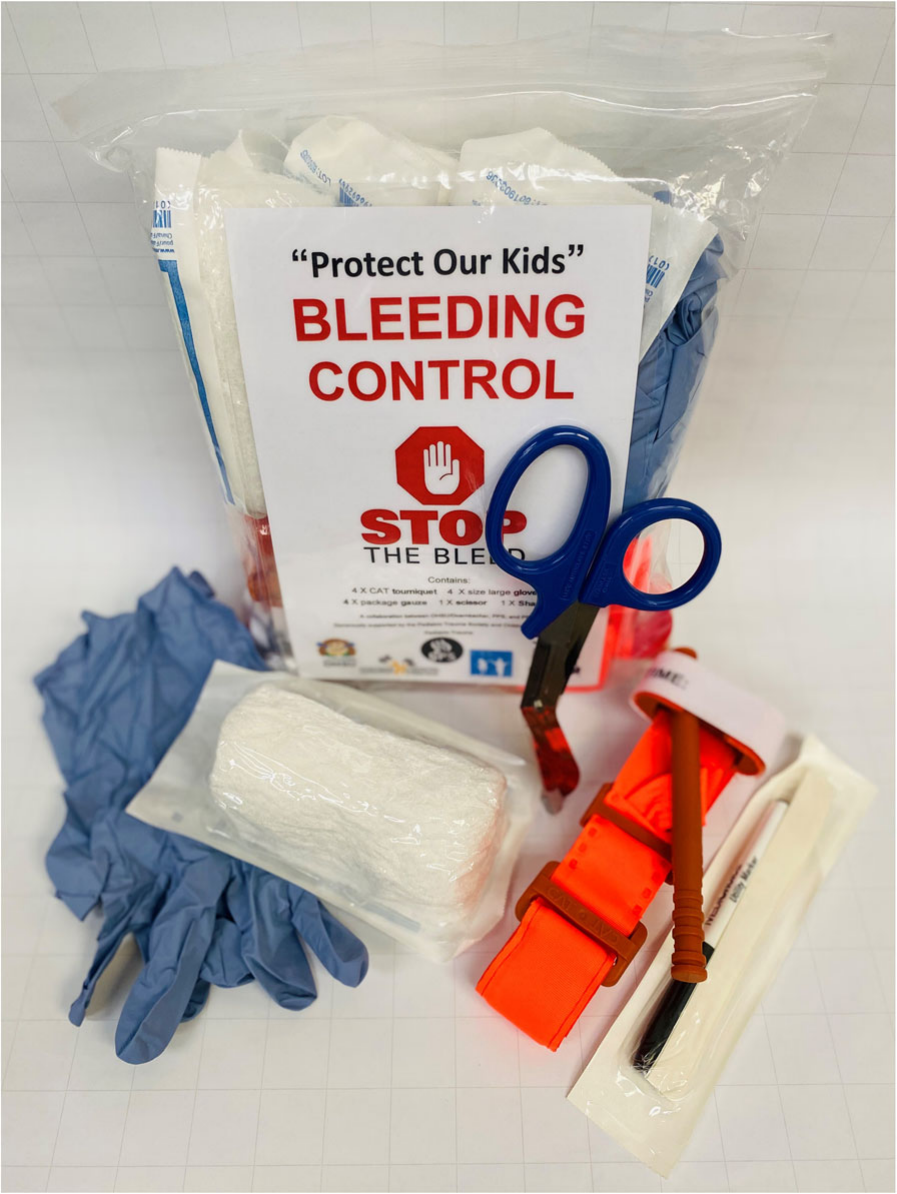
Hemorrhage control kits fabricated by the OHSU Trauma Program from materials purchased in bulk – $122/kit: 4 x North American Rescue Combat Tourniquets – $29/tourniquet. 4 x large gloves. 4 x packages of gauze. 1 x utility scissor. 1 x surgical marker

One thousand eighteen school staff participated in Protect Our Kids at 25 elementary and middle schools. 78.2% of participants were teachers, 5.8% were para-educators such as teacher’s aides and classroom assistants, 4.4% were counselors and 3.4% were administrators (Fig. 1). Eighteen trainers participated in the program: six physicians, six nurses and six police officers. Ninety-five percent of school staff rated the program as being very important on an individual basis and 94% rated the program as being very important to the public at large. Seventy-four percent of school staff reported feeling well-prepared to assist in a bleeding emergency and 89% thought that the program was very effective in teaching lifesaving skills during a critical incident (Fig. 2). Seventy-five hemorrhage control kits were dispensed with North American Rescue Combat Application Tourniquets representing the bulk of expense at a cost of $29 per tourniquet (Fig. 3). Each school received three kits. All trainers rated the program as being very effective and valuable. All trainers would volunteer to participate in the future.

Salient positive feedback included: “I appreciate the brevity of this training—I learned simple steps that can help”, “I am so thankful for this opportunity” and “I feel confident that I can do this if confronted with bleeding.” Constructive feedback included: “I think it is impossible to know how to respond after something so terrible” and “we should have periodic retraining so that these procedures become more automatic.”

## Discussion

Preventing mortality from active shooter events in schools is a complex, politically contested issue that requires changes in gun safety policies, earlier recognition of warning signs in perpetrators, effective mental health services, school security upgrades and optimal trauma care. One important effort is disseminating Stop the Bleed in schools across the nation. Thus far, the state of Georgia has launched Stop the Bleed in all its public schools and placed hemorrhage control kits in all buildings (Stop the Bleed Georgia 2020). School-based Stop the Bleed initiatives have also appeared in Pennsylvania, Florida and Texas, among other states (Bolleter 2016; Gomez 2018; Neal et al. 2018). Recent work has shown that high schoolers and children as young as sixth-graders can be effectively taught hemorrhage control (Sidwell et al. 2020; Goolsby et al. 2020). Unlike first aid and CPR, however, Stop the Bleed has yet to become standard or even mandatory for school staff in most states, nor are hemorrhage control kits as widely available or legally regulated as AEDs.

This study illustrates the importance of collaboration between trauma centers, law enforcement and schools, and the feasibility, ease and effectiveness of administering Stop the Bleed to school staff. The ability to deliver this program to over 1000 educators serving a population of almost 10,000 students is a model for other trauma programs. The initial investment in hemorrhage control kits is a minor expense compared to the potential number of lives saved.

One limitation of the study is a lack of long-term follow-up to evaluate the effectiveness of hemorrhage control training over time. Although techniques taught in Stop the Bleed are straightforward, skills acquired may decline over time. Further study to determine the degree of content retention would permit recommendations around the timing of recertification. A second limitation is application of this program to a single geographical area, which makes generalizability difficult to interpret. Given the ease of program administration and overwhelmingly positive response, it is reasonable to expect similar results in other settings. Lastly, the unforeseen challenge of the COVID-19 pandemic has limited further deployment of Protect Our Kids to the remainder of the state. Future efforts will require delivering training in a physically distanced environment. The American College of Surgeons Committee on Trauma recently added surgical masks to hemorrhage control kits and published suggestions for how to safely deliver Stop the Bleed training during the COVID-19 pandemic (American College of Surgeons 2020).

## Conclusions

This study reports on a novel initiative to disseminate Stop the Bleed to elementary and middle schools in a large metropolitan area. Our work underscores the need for widespread hemorrhage control training and access to bleeding control kits. The ultimate goal must be to classify hemorrhage as a public health threat and to increase the comfort and willingness of the lay public to intervene in a manner similar to bystander CPR.

## Data Availability

The datasets used and analyzed in the study are available from the corresponding author upon reasonable request.

## Abbreviations

PPB: Portland Police Bureau
OHSU: Oregon Health & Science University
CPR: Cardiopulmonary resuscitation
AED: Automatic external defibrillator
